# Coronary stent imaging in photon counting computed tomography: improved imaging of in-stent stenoses in a phantom with optimized reconstruction kernels

**DOI:** 10.1093/bjro/tzae030

**Published:** 2024-10-18

**Authors:** Arwed Elias Michael, Denise Schoenbeck, Jendrik Becker-Assmann, Nina Pauline Haag, Julius Henning Niehoff, Bernhard Schmidt, Christoph Panknin, Matthias Baer-Beck, Tilman Hickethier, David Maintz, Alexander C Bunck, Roman Johannes Gertz, Jan Borggrefe, Jan Robert Kroeger

**Affiliations:** Department of Radiology, Neuroradiology and Nuclear Medicine, Johannes Wesling University Hospital, Ruhr University Bochum, 44801 Bochum, Germany; Department of Radiology, Neuroradiology and Nuclear Medicine, Johannes Wesling University Hospital, Ruhr University Bochum, 44801 Bochum, Germany; Department of Radiology, Neuroradiology and Nuclear Medicine, Johannes Wesling University Hospital, Ruhr University Bochum, 44801 Bochum, Germany; Department of Radiology, Neuroradiology and Nuclear Medicine, Johannes Wesling University Hospital, Ruhr University Bochum, 44801 Bochum, Germany; Department of Radiology, Neuroradiology and Nuclear Medicine, Johannes Wesling University Hospital, Ruhr University Bochum, 44801 Bochum, Germany; Siemens Healthineers AG, 91301 Forchheim, Germany; Siemens Healthineers AG, 91301 Forchheim, Germany; Siemens Healthineers AG, 91301 Forchheim, Germany; Institute for Diagnostic and Interventional Radiology, Faculty of Medicine and University Hospital Cologne, University of Cologne, 50931 Cologne, Germany; Institute for Diagnostic and Interventional Radiology, Faculty of Medicine and University Hospital Cologne, University of Cologne, 50931 Cologne, Germany; Institute for Diagnostic and Interventional Radiology, Faculty of Medicine and University Hospital Cologne, University of Cologne, 50931 Cologne, Germany; Institute for Diagnostic and Interventional Radiology, Faculty of Medicine and University Hospital Cologne, University of Cologne, 50931 Cologne, Germany; Department of Radiology, Neuroradiology and Nuclear Medicine, Johannes Wesling University Hospital, Ruhr University Bochum, 44801 Bochum, Germany; Department of Radiology, Neuroradiology and Nuclear Medicine, Johannes Wesling University Hospital, Ruhr University Bochum, 44801 Bochum, Germany

**Keywords:** computed tomography, photon counting computed tomography, photon counting detector, in-stent stenosis, stent imaging

## Abstract

**Objectives:**

Coronary CT angiography (CCTA) is becoming increasingly important in the workup of coronary artery disease. Imaging of stents and in-stent stenoses remains a challenge. This work investigates the assessability of in-stent stenoses in photon counting CT (PCCT) using ultra-high-resolution (UHR) imaging and optimized reconstruction kernels.

**Methods:**

In an established phantom, 6 stents with inserted hypodense stenoses were scanned in both standard resolution (SRM) and UHR in a clinical PCCT scanner (NAEOTOM Alpha, Siemens Healthineers, Germany). Reconstructions were made both with the clinically established and optimized kernels. The visible stent lumen and the extent of stenosis were quantitatively measured and compared with the angiographic reference standard. Also, region-of-interest (ROI)-based measurements and a qualitative assessment of image quality were performed.

**Results:**

The visible stent lumen and the extent of stenosis were measured more precisely in UHR compared to SRM (0.11 ± 0.19 vs 0.41 ± 0.22 mm, *P* < .001). The optimized kernel further improved the accuracy of the measurements and image quality in UHR (0.35 ± 0.23 vs 0.47 ± 0.19 mm, *P* < .001). Compared to angiography, stenoses were overestimated in PCCT, on average with an absolute difference of 18.20% ± 4.11%.

**Conclusions:**

Photon counting CCTA allows improved imaging of in-stent stenoses in a phantom using UHR imaging and optimized kernels. These results support the use of UHR and optimized kernels in clinical practice and further studies.

**Advances in knowledge:**

UHR imaging and optimized reconstruction kernels should be used in CCTA in the presence of cardiac stents.

## Introduction

Coronary artery disease (CAD) is highly prevalent and associated with significant mortality and economic burden.^1^ Coronary CT angiography (CCTA) is an increasingly important component of non-invasive diagnostics of CAD. While CCTA was initially primarily used to rule out CAD in patients with a low pre-test probability, the range of indications has expanded significantly in recent years. CCTA is increasingly being used for re-evaluation in patients with known CAD, and now also for planning coronary interventions.^2^ As a result, more and more patients with coronary stents are being examined. In those examinations, the lumen of the stents must be assessed to evaluate a possible in-stent stenosis. However, assessment of in-stent stenosis is challenging. Even with third-generation dual-source CT, false-positive findings persist in 25% of cases, and the in-stent lumina are often not adequately assessed.^3^ With photon counting CT (PCCT) now being used in clinical practice, technical advances are available that also have an impact on CCTA^4^^,^^5^ and may also improve the assessability of in-stent stenoses. Initial studies show that improved image quality and diagnostic confidence can be achieved in PCCT compared to conventional CT detectors^6^; also, improved characterization of plaques is possible.^7^

Studies on the assessability of in-stent stenoses, however, are currently available only with a preclinical PCCT. The promising results showed that the measurement of the in-stent lumen and in-stent stenoses could be improved.^8^ Although many authors believe that photon counting detector technology offers great potential for advancing image quality, it is sometimes still limited by artefacts.^9^ In general, the need for dedicated post-processing algorithms for artefact reduction remains, even with photon counting detector technology. A recent study has shown that the assessment of the in-stent lumen can be significantly improved with optimized reconstruction kernels by reducing artefacts.^10^ This study will consequently investigate whether the optimized reconstruction kernels can also improve the assessability of in-stent stenoses. Using an established model and the clinically available PCCT, the different resolution modes as well as the clinical and new optimized reconstruction kernels are examined.

## Methods 

### Phantom of in-stent stenosis

A pre-existing model was used to evaluate in-stent stenoses.^8^ In this model, a tube made of synthetic material with a density of 30 HU and an inner diameter of 3 mm represented the coronary vessel. The wall thickness of the synthetic tube was about 0.3 mm. Six different stents of different materials and strut thicknesses (see Table 1) were selected. The stent materials cobalt chromium, steel, nitinol, tantalum, and platinum chromium represent common stent metals. Stents with gold coatings were excluded, as previous studies have shown that massive artefacts still prevent the assessment of the in-stent lumen in PCCT.^10^ All stents were implanted regularly a few years ago and therefore might occur in patients who undergo a re-evaluation of CAD and stent patency using CCTA. The stents were placed in the centre of the synthetic tubes. The artificial hypodense stenoses were made of a wax-based material mixed with a lipophilic contrast agent (Lipiodol Ultra-Fluid; Guerbet GmbH, Sulzbach, Germany) titrated to measure 45 HU at 120 kVp.^8^ A portion of this material was angiographically positioned in the lumen of the stent. The material was then pressed and thus fixed to the stent strut with a 1.5-mm balloon catheter (Armada 14; Abbott GmbH, Wiesbaden, Germany) over a microwire (V-14 Control Wire; Boston Scientific GmbH, Ratingen, Germany). For a schematic representation, see also Figure S1.

**Table 1.** Stent characteristics.

Name	Manufacturer	Material	Diameter (mm)	Length (mm)	Strut thickness (mm)
Chrono	Sorin Biomedical	CoCr	3	20	0.08
Coroflex Please	Braun	StSt 316 L	3	19	0.12
Endeavor	Medtronic	CoCr	3	30	0.091
Promus Element Plus	Boston Scientific	PlCr	3	19	0.081
Radius	Boston Scientific	Nitinol	3	20	0.085
Tantal Coronary	Abbott/Guidant	Tantalum	3	19	0.058

Abbreviations: CoCr = cobalt chromium; PlCr = platinum chromium; StSt = stainless steel.

### Determination of ground truth

Quantification of the generated stenoses was performed angiographically according to the clinical gold standard. For this purpose, the tubes were filled with undiluted contrast medium (300 mg iohexol/mL, Accupaque 300, GE Healthcare, Chicago, IL, United States), sealed airtight, and placed in *z*-axis on the angiography table. Using rotational angiography, projections were acquired at every angle. The projection with the angiographically largest residual lumen adjacent to the artificial plaque was selected. A calibrated electronic measurement tool was then used to measure the diameter of the tube and stent, including the stent struts. Also, the diameter of the plaque (with stent struts adjacent on one side) and the diameter of the residual lumen (with stent struts adjacent on one side) were determined for each stenosis (see Table 2).

**Table 2.** Ground truth of in-stent stenosis.

Stent types	Tube diameter	Stent diameter	Lumen measured	Plaque measured	Strut thickness	True lumen stent	True lumen stenosis	True plaque	Stenosis (%)
Chrono	2.80	2.80	0.90	1.90	0.080	2.640	0.820	1.82	68.94
Coroflex Please	2.80	2.80	1.20	1.60	0.120	2.560	1.080	1.48	57.81
Endeavor	2.80	2.80	0.90	1.90	0.091	2.618	0.809	1.81	69.10
Promus Element Plus	3.00	3.00	1.00	2.00	0.081	2.838	0.919	1.92	67.62
Radius	2.80	2.80	1.30	1.50	0.085	2.630	1.215	1.42	53.80
Tantal Coronary	2.60	2.60	0.90	1.70	0.058	2.484	0.842	1.64	66.10

All measurements given in (mm), except the relative diameter stenosis (%). The stent diameter, lumen, and plaque were measured angiographically including the stent strut; the true lumen and true plaque diameter are equal to the measured diameter minus the strut thickness.

### CT protocol and image acquisition

The vessel model was filled with a solution of saline (0.9%) and contrast agent (300 mg iohexol/ml, Accupaque 300, GE Healthcare), adjusted so that it measured 600 HU in a polyenergetic reconstruction of a scan with 120 kVp in the PCCT. According to our experience, this density provides optimal contrast in CCTA and can be regularly achieved in clinical examinations. The tube was then sealed airtight on both sides and placed in a saline-filled plastic container measuring (length) 36 cm × (width) 24 cm × (height) 14 cm (see Figure S1). This container was placed parallel to the *z*-axis in the gantry of the scanner with the stent being slightly below the isocentre.

The phantom was examined in the dual source PCCT (NAEOTOM Alpha, software version syngo CT VA50, Siemens Healthineers, Germany) with a sequential CT protocol with a tube voltage of 120 kVp and an effective tube current of 50 mAs, matching the average tube current of CCTA examinations performed in our clinical routine. For each stent, 2 scans were performed: one in standard resolution (SRM) with a total collimation of 144 × 0.4 mm and the focal spot size of 0.8 × 1.1 mm. In SRM, the Quantumplus mode allowed for the acquisition of spectral data and the calculation of virtual monoenergetic images (VMIs). The other scan was performed using ultra-high-resolution (UHR) with a total collimation of 120 × 0.2 mm and a focal spot size of 0.6 × 0.7 mm. For the UHR mode, the acquisition of spectral data was not available at the time of the experiments. With these settings, 2 raw data sets were created for each stent.

### Kernels and reconstructions

**Figure 1.**
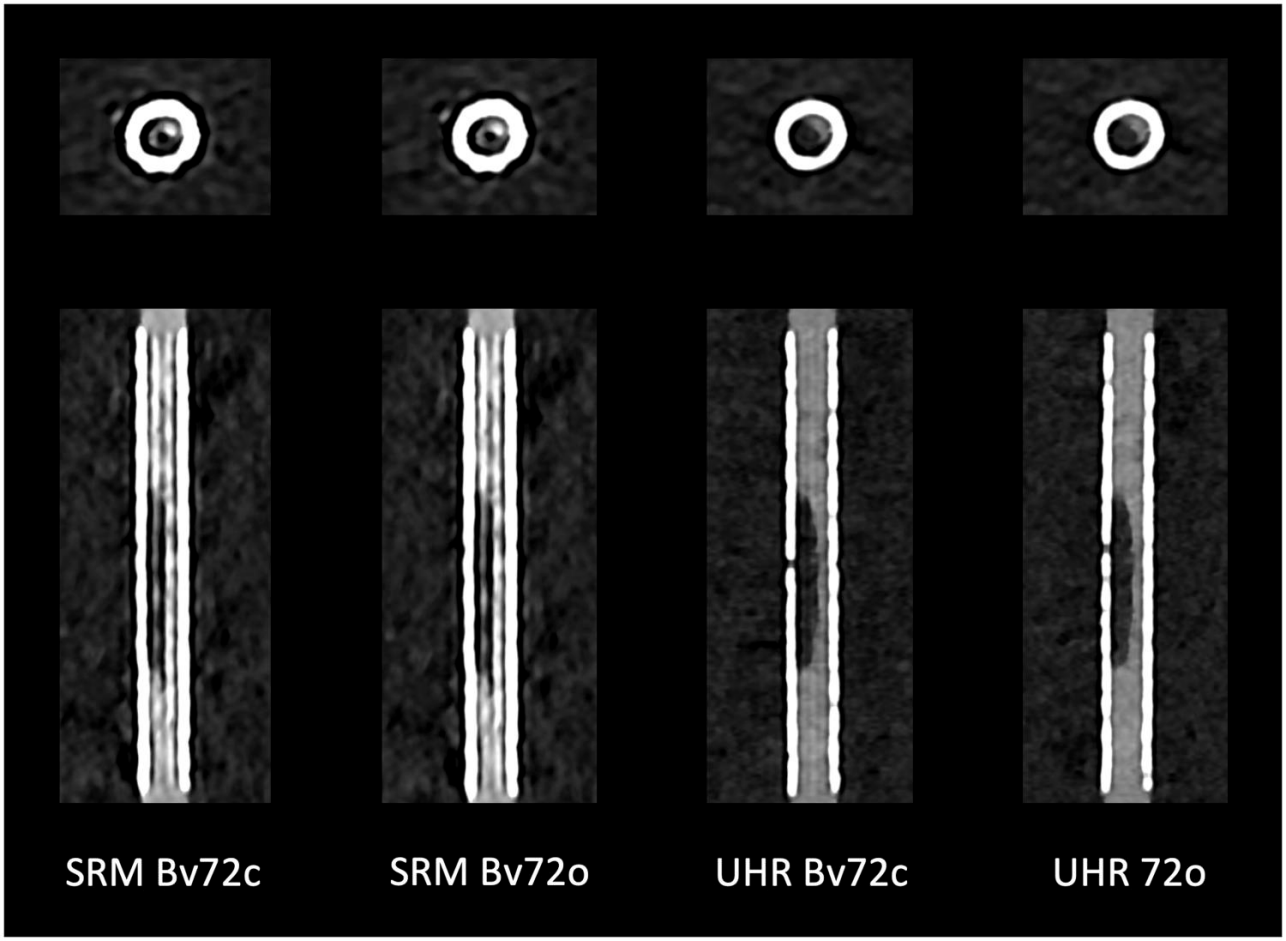
Optimization of reconstruction kernels. Based on experimental data, vascular reconstruction kernels were improved with respect to stent imaging. In doing so, the extent of artefacts inside the stent lumina was successfully reduced. Note the reduced hyperdense streak artefacts. Abbreviations: Bv72c = clinically established vascular reconstruction kernel Bv72; Bv72o = optimized kernel; SRM = standard resolution mode; UHR = ultra-high-resolution mode.

Four primary reconstructions were made for each stent based on the aforementioned data sets in SRM and UHR with minimal slice thickness (see Table 3) using the clinically established kernel Bv72 (Bv72c) and this kernel optimized for imaging of coronary stents (Bv72o). The process of optimization (see Figure 1) of the kernels was based on experimental investigations in our institute.^10^ Reconstructions were computed on a workstation with a preclinical reconstruction software (ReconCT 16.0.0.2718_PRERELEASE), since the preclinical optimized kernel could not be used on the computing unit of the CT scanner. The field of view was narrowed down to approximately 65 × 65 mm, so that the stent was fully imaged with surrounding saline on every side. For all reconstructions and VMI, the third level of iterative reconstruction (Quantum Iterative Reconstruction, 4 levels of iteration), as this is currently done in our clinical routine. Strictly axial and longitudinal multiplanar reconstructions (MPRs) of each in-stent stenosis were reconstructed for further analysis using the clinical CT spectral workstation (syngo.via, version VB60_B). In those MPRs, the slice thickness was set to 0.1 mm and the increment to 0.1 mm.

**Table 3.** Reconstructions for each stent.

Reconstruction name	Resolution	Kernel	MonoE/PER	Slice thickness (mm)	Increment (mm)	Matrix	Iteration
SRMc	SRM	Bv72fc	MonoE 55 keV	0.4	0.2	1024 × 1024	3
SRMo	SRM	Bv72fo	MonoE 55 keV	0.4	0.2	1024 × 1024	3
UHRc	UHR	Bv72uc	PER	0.2	0.2	1024 × 1024	3
UHRo	UHR	Bv72uo	PER	0.2	0.2	1024 × 1024	3

Abbreviations: Bv72fc = currently clinically used kernel Bv72 for SRM; Bv72fo = optimized kernel Bv72 for SRM; Bv72uc = currently clinically used kernel Bv72 for UHR; Bv72uo = optimized kernel Bv72 for UHR; keV = kilo-electron volt; MonoE = virtual monoenergetic reconstruction; PER = polyenergetic reconstruction; SRM = standard resolution mode; UHR = ultra-high-resolution mode.

### Image analysis

Five readers performed the image analysis. Readers 1 and 2 were radiological residents with 3 years and 1 year of experience in cardiac CT imaging. Reader 3 was a radiological consultant with 10 years, Reader 4 was a radiological resident with 2 years, and finally, Reader 5 was a resident with 1 year of experience in cardiac CT imaging. The quantitative measurements of the angiographic ground truth stent images were carried out separately by an experienced radiological consultant. All readers were blinded to information regarding the stent material and the amount of stenosis.

The analysis of the images was based on already established methods^8^^,^^11^ using fixed window settings (C600 W1500).^8^^,^^12^ The first 2 readers performed ROI-based measurements: in the axial reconstruction, the first ROI was set in the contrast-filled synthetic vessel model beyond the stent material, the second ROI was placed in the visible stent lumen prior to the stenosis, the third ROI in the plaque, and finally, the fourth ROI in the saline in the plastic container surrounding the stent. The mean CT value and its SD were documented for every ROI. The SD of the CT value of the surrounding saline was defined as the general noise. The contrast-to-noise ratio (CNR) of stent lumen and plaque was defined as the difference of the mean CT value of the stent lumen and the plaque divided by the square root of the sum of the CT value variances.

All readers measured the visible lumen in the stent proximal to the stenosis, the visible residual lumen diameter in the stenosis as well as the plaque diameter in the axial and longitudinal reconstruction. For qualitative analysis, the assessability of the in-stent stenosis was rated on a 5-point Likert scale (1: massive artefacts, stenosis not assessable; 5: hardly any artefacts, stenosis well assessable). Finally, the readers were asked to designate the reconstruction that was most helpful in assessing the stenoses (axial or longitudinal).

### Statistical analysis

The statistical analysis software R (Version 4.1.0^13^) and RStudio (Version 2022.07.1 + 554) were used for data curation, data processing, and statistical analysis. Hierarchical testing had been determined prior to data acquisition. It was planned to first compare UHR to SRM, then the comparison of the reconstructions using the clinical and optimized kernels. The Shapiro-Wilk test and the Levene test were applied to check for value distribution and homoscedasticity. One-way ANOVA, or the Friedman test, as the corresponding non-parametric method, was used for the comparison of means; *t*-test and Wilcoxon test were used in *post hoc* analysis. The binomial test was used to analyse the distribution of dichotomous nominal variables. The Bonferroni method was applied to correct *P* values. The intraclass correlation coefficient (ICC) and Fleiss kappa were used to calculate inter-reader reliability. Data are presented as mean ± SD, if not explicitly stated otherwise.

## Results

### ROI-based measurements

The increase in density in the lumen of the stent compared with the density in the contrasted tube outside the stent was 132.33 ± 178.07 HU in the reconstruction in SRM with the clinical kernel (SRMc), and 139.25 ± 162.69 HU in the reconstruction in SRM with the optimized kernel (SRMo). In comparison, the increase in density in the reconstruction using UHR with the clinically established kernel (UHRc) was 35.92 ± 126.46 HU, in the reconstruction with the optimized kernel (UHRo) 56.25 ± 154. The differences between SRM and UHR considering both kernels proved to be significant (corrected *P* = .045). There was no significant difference between UHRc and UHRo (corrected *P* = .273).

The noise was 75.16 ± 86.81 HU in SRMc and 55.67 ± 13.30 HU in SRMo. In UHRc, the noise was 62.58 ± 46.36 HU, in UHRo, 39.75 ± 8.35 HU. The difference between SRM and UHR again was significant (corrected *P* = .039), but not between the clinical and optimized kernel (corrected *P* = .204).

For CNR between the stent lumen and plaque of the stenosis, 1.49 ± 0.46 was achieved in SRMc, and 1.99 ± 0.63 in SRMo. In UHR, CNR increased significantly to 5.19 ± 2.63 in UHRc and 5.61 ± 2.54 in UHRo. Again, the difference between SRM and UHR was significant (corrected *P* < .001), but not between UHRc and UHRo (corrected *P* = .388).

### Visible in-stent lumen

**Figure 2.**
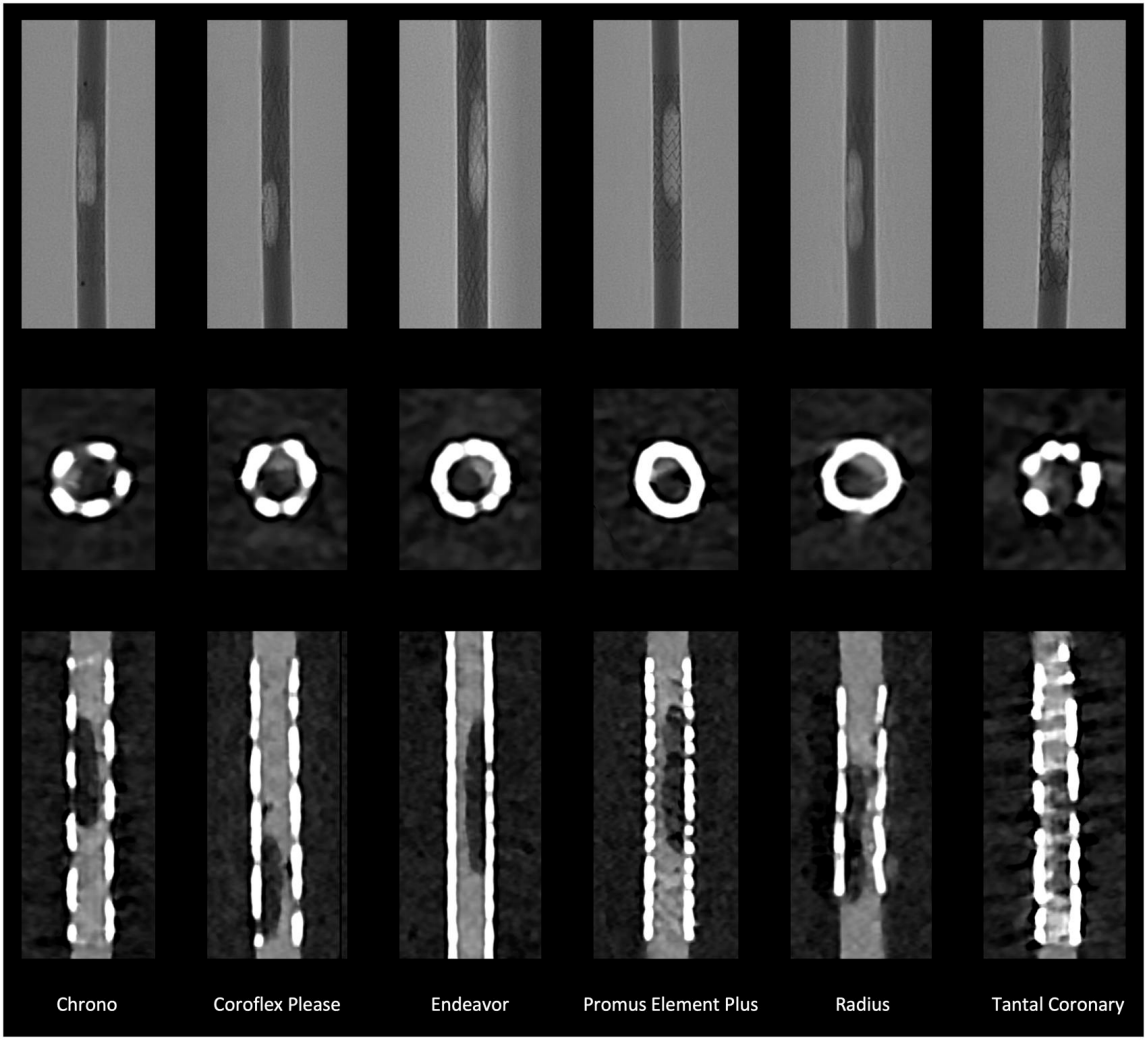
Angiography and PCCT of in-stent stenosis. The first row shows the angiographic image of the stents with the stenoses in optimal projection for each stent used. The second row shows the axial reconstruction, the third the corresponding longitudinal reconstruction of the CCTA. CT images reconstructed with UHR data sets and the optimized Bv72 kernel. Abbreviations: PCCT = photon counting CT; UHR = ultra-high-resolution mode.

The visible lumen averaged 0.27 ± 0.49 mm in SRM (in most cases, no part of the lumen could be adequately assessed and was thus measured at 0 mm) and 1.37 ± 0.55 mm in UHR (corrected *P* < .001). In UHRc, the visible lumen averaged 1.32 ± 0.57 mm, and in UHRo, it was 1.43 ± 0.54 mm (corrected *P* = .033). Figure 2 shows the visibility of the stent lumen and stenosis in the optimal reconstruction (UHRo).

### Assessment of in-stent stenosis

**Figure 3.**
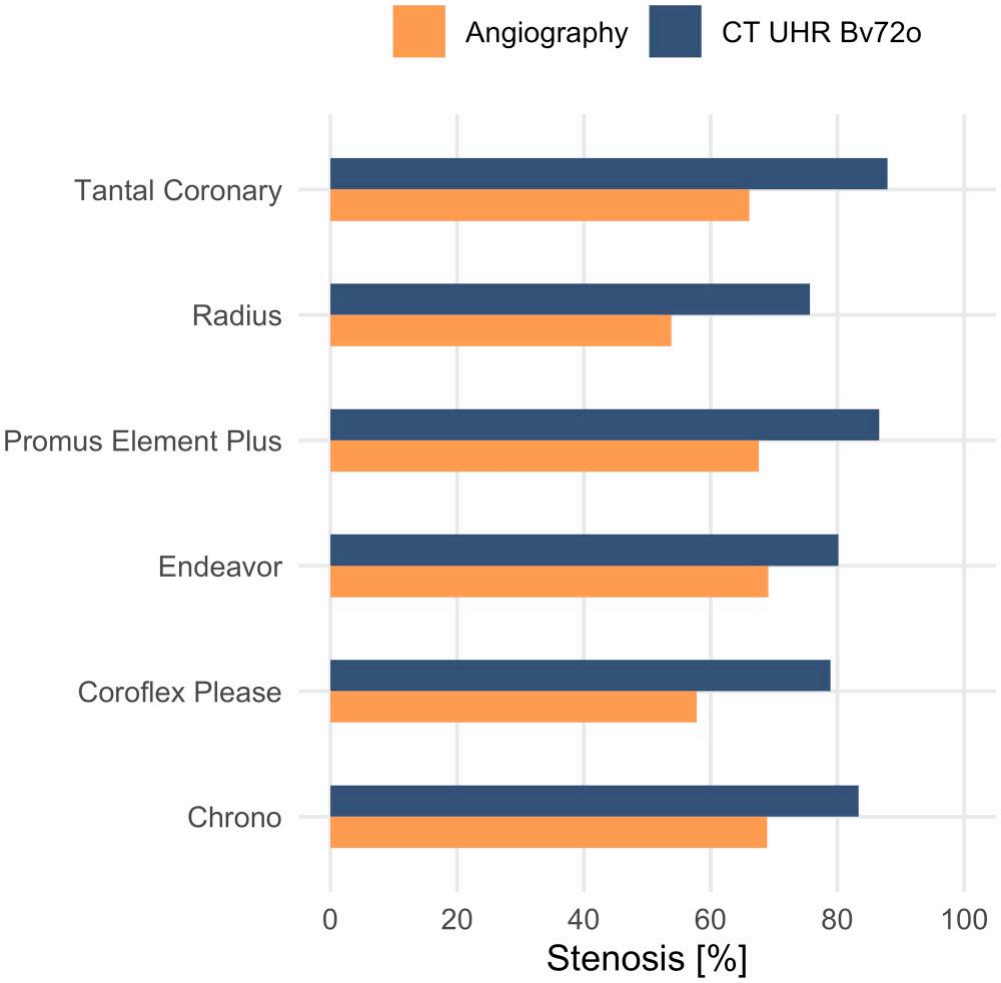
Comparison of angiographic (gold standard) and PCCT CCTA measurement of in-stent stenosis. The stenosis is reported in percentage of the area (%) for each stent. In CCTA, any stenosis is overestimated. Abbreviations: Bv72o = optimized vascular reconstruction kernel Bv72; CCTA = coronary CT angiography; PCCT = photon counting CT; UHR = ultra-high-resolution mode.

The intraclass correlation showed a fair inter-reader reliability (ICC = 0.47). The residual lumen was measured more accurately in UHR than in SRM (mean residual lumen in SRM was 0.11 ± 0.19 mm vs in UHR 0.41 ± 0.22 mm, corrected *P* < .001). The difference between UHRc and UHRo also proved to be significantly in favour of UHRo (UHRc 0.35 ± 0.23 mm vs UHRo 0.47 ± 0.19 mm, corrected *P* = .021). There was no difference between the readings when using axial and longitudinal reconstruction (corrected *P* = .818). However, the readers indicated that longitudinal reconstruction was more helpful in assessing stenosis (corrected *P* < .001). For each stent, UHRo also overestimated the stenosis (see Figure 3 and Table 4).

**Table 4.** Angiographic and PCCT CCTA measurement of in-stent stenosis.

Stent	Stenosis CCTA (%)	Stenosis angiography (%)	Difference (%)
Chrono	83.33	68.94	14.39
Coroflex Please	78.91	57.81	21.1
Endeavor	80.14	69.10	11.04
Promus Element Plus	86.61	67.62	18.99
Radius	75.67	53.80	21.87
Tantal Coronary	87.92	66.10	21.82

In each case, diameter stenosis is given in %. The difference is given as the absolute difference in %. The absolute difference is 18.20% ± 4.11%.

Abbreviations: CCTA = coronary CT angiography; PCCT = photon counting computed tomography.

**Figure 4.**
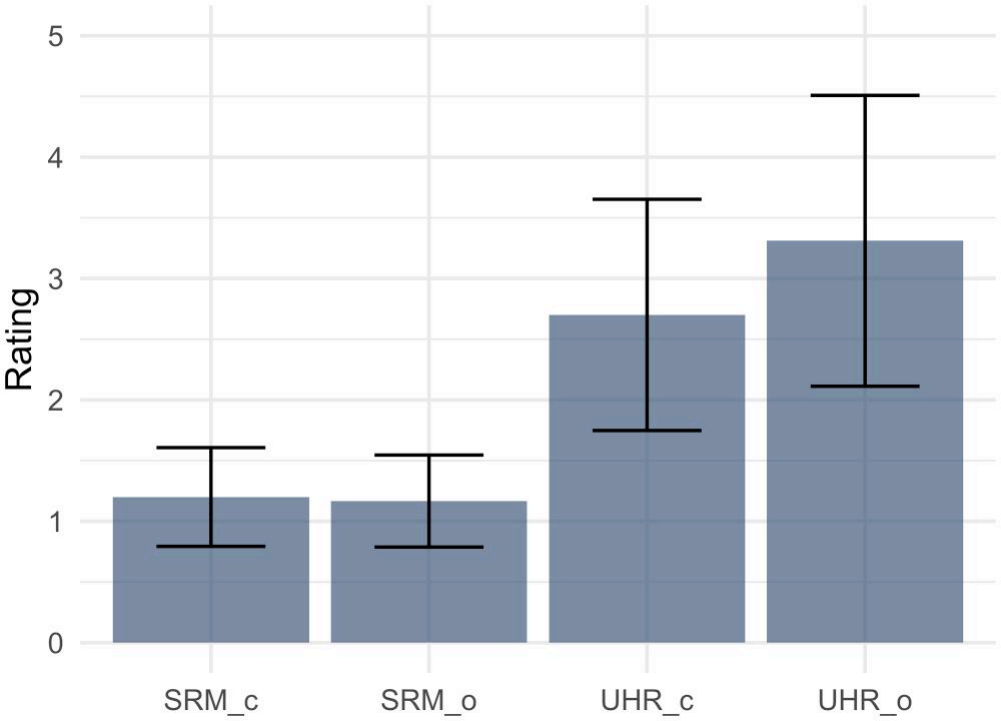
Rating of the 4 different reconstructions in terms of assessability of in-stent stenosis. Abbreviations: c = clinically established reconstruction kernel Bv72; Likert Scale (1: massive artefacts, stenosis not assessable; 5: hardly any artefacts, stenosis well assessable); o = optimized reconstruction kernel Bv72; SRM = standard resolution mode; UHR = ultra-high-resolution mode.

In the qualitative rating, there was only fair inter-rater reliability (Fleiss κ = 0.31). In comparison of all reconstructions, UHRo received the highest rating regarding the assessability of the stenosis (3.31 ± 1.20, see Figure 4). The difference between SRM and UHR and between UHRc and UHRo was significant (for both comparisons, corrected *P* < .001).

## Discussion

To the best of our knowledge, this is the first investigation of in-stent stenosis with optimized reconstruction kernels. Our results show that PCCT with UHR and optimized kernels allowed for assessment of in-stent stenosis in coronary stents in a phantom. Both the residual stent lumen outside the stenosis and the residual lumen diameter in the region of the stenosis could be determined more accurately in UHR with optimized kernels. The image quality is also significantly improved. However, even with optimal reconstruction parameters, all stenoses were still overestimated in PCCT compared to angiography, with an average overestimation of 18.20% ± 4.11%.

CAD is the leading cause of mortality in many developed countries and poses significant morbidity and economic burden.^14^ Reduction in cardiovascular risk factors, optimized medical treatment, and surgical bypass grafts are important pillars in the treatment of CAD, but coronary artery interventions, especially coronary artery stenting, are increasingly used to treat coronary artery stenoses in acute and chronic CAD, with more than 600 000 stents implanted annually in the United States.^15^ CCTA is increasingly adopted as the primary diagnostic tool to evaluate the coronary arteries according to current guidelines,^16^ with invasive coronary angiography being reserved for unclear and interventional cases. Thus, an increasing number of patients with coronary stents is presenting for CCTA. Even though the use of drug-eluting stents has reduced the rate of in-stent restenosis to 5%-15% compared to bare metal stents,^17^ in-stent restenosis is still an important clinical problem. CCTA with modern CT scanners allows for a rule-in/rule-out of significant in-stent restenosis in many patients with coronary artery stents.^18^ However, exact quantification of stenosis remains challenging, due to different types of artefacts.^19^

The introduction of PCCT into the clinical routine has already shown improvement in a vast spectrum of applications,^20^ with the absence of electronic noise, UHR capability, and improved iodine CNR being most important for CCTA. However, literature regarding the use of PCCT for the identification of in-stent stenosis is still sparse. For instance, Boccalini et al^21^ have shown first in-human results using a preclinical PCCT. In their study, they examined 8 patients with a total of 16 stents using a state-of-the-art CT with energy-integrating dual layer detector (EID-DLCT) and a preclinical PCCT. Five stents could not be assessed due to motion artefacts, while the remaining stents showed improved visualization in the PCCT compared to the EID-DLCT. By reducing the artefacts, a larger proportion of the in-stent lumen could be measured, and the outer diameter was smaller compared to the EID-DLCT. The CT values in the contrasted vessel outside and inside the stent also showed smaller differences. The image quality of the PCCT was also rated as superior in the subjective assessment. Comparable to the results of our study, a sharp reconstruction core achieved the best results.^21^ Decker et al^22^ have shown phantom results using the clinical PCCT and were able to show that UHR mode allows for significantly improved in-stent lumen visibility. Consistent with other recent studies,^10^ they were able to show that a sharper reconstruction kernel is the best to assess the in-stent lumen in small stents. However, they did not evaluate in-stent stenosis.^22^ The vascular reconstruction kernel used in their study (Bv kernel) was the same as the clinical kernel used in our experiment and was the starting point for kernel optimization.^10^ Finally, Bratke et al^8^ evaluated a preclinical PCCT prototype using a phantom with in-stent stenosis in cardiac stents similar to the phantom in our study. This study also primarily compared the PCCT with the EID-DLCT in terms of the assessability of in-stent stenoses. The in-stent lumen and also the stenosis could be measured significantly better in the PCCT. Using EID-DLCT, the stenoses could not be delineated in any of the stents used, whereas in the PCCT, measurements could be carried out in 7 out of 10 stents. This resulted in an average measured stenosis of around 65%, meaning that the actual 50% stenosis was overestimated by around 15%.^8^ Compared to Bratke et al,^8^ we used the now available clinical PCCT and employed a novel reconstruction kernel designed especially for imaging of small-diameter stents. Our results showed a significantly improved assessability of the in-stent lumen using UHR compared to SRM. The optimized kernel also improved subjective and objective image quality. Applying the best image reconstruction kernel led to an overestimation of the in-stent stenosis by 18% on average when compared to angiography, comparable to the study of Bratke et al.^8^ A trend to overestimate stenoses compared to angiography is often seen in CCTA but still allows CCTA to fulfil its role as a rule-out modality, potentially sparing patients an invasive angiography procedure.

Our study has several limitations. We only performed experiments in a simple phantom that is obviously very different from a real patient. Especially, we did not simulate cardiac motion that might reduce image quality extensively. Moreover, our stenoses consisted of hypodense material without any calcifications in the plaque or vessel wall, which would have posed additional challenges. Finally, the used optimized reconstruction kernel is a research prototype and not yet available for clinical routine.

In conclusion, PCCT with UHR and optimized reconstruction kernels allows for an effective evaluation of in-stent stenosis in coronary stents in a phantom model. Our results provide guidance for reconstruction of CCTA data sets in patients with coronary stents, and future studies should aim to prove the effectiveness of PCCT for in-stent stenosis *in vivo*.

## Supplementary Material

tzae030_Supplementary_Data
