# Supplementary material for: Coronary stent imaging in photon counting computed tomography: improved imaging of in-stent stenoses in a phantom with optimized reconstruction kernels
Source: BJR Open. 2024 Oct 18;6(1):tzae030. doi: 10.1093/bjro/tzae030 (PMC11498892; doi:10.1093/bjro/tzae030)
Supplement: tzae030_Supplementary_Data [file tzae030_supplementary_data.docx]

Supplementary Material

Supplementary figure for the original research article with the title

**Coronary Stent Imaging in Photon Counting Computed Tomography: Improved Imaging of In-Stent Stenoses in a Phantom with Optimized Reconstruction Kernels**

**Schematic Representation of the Phantom**


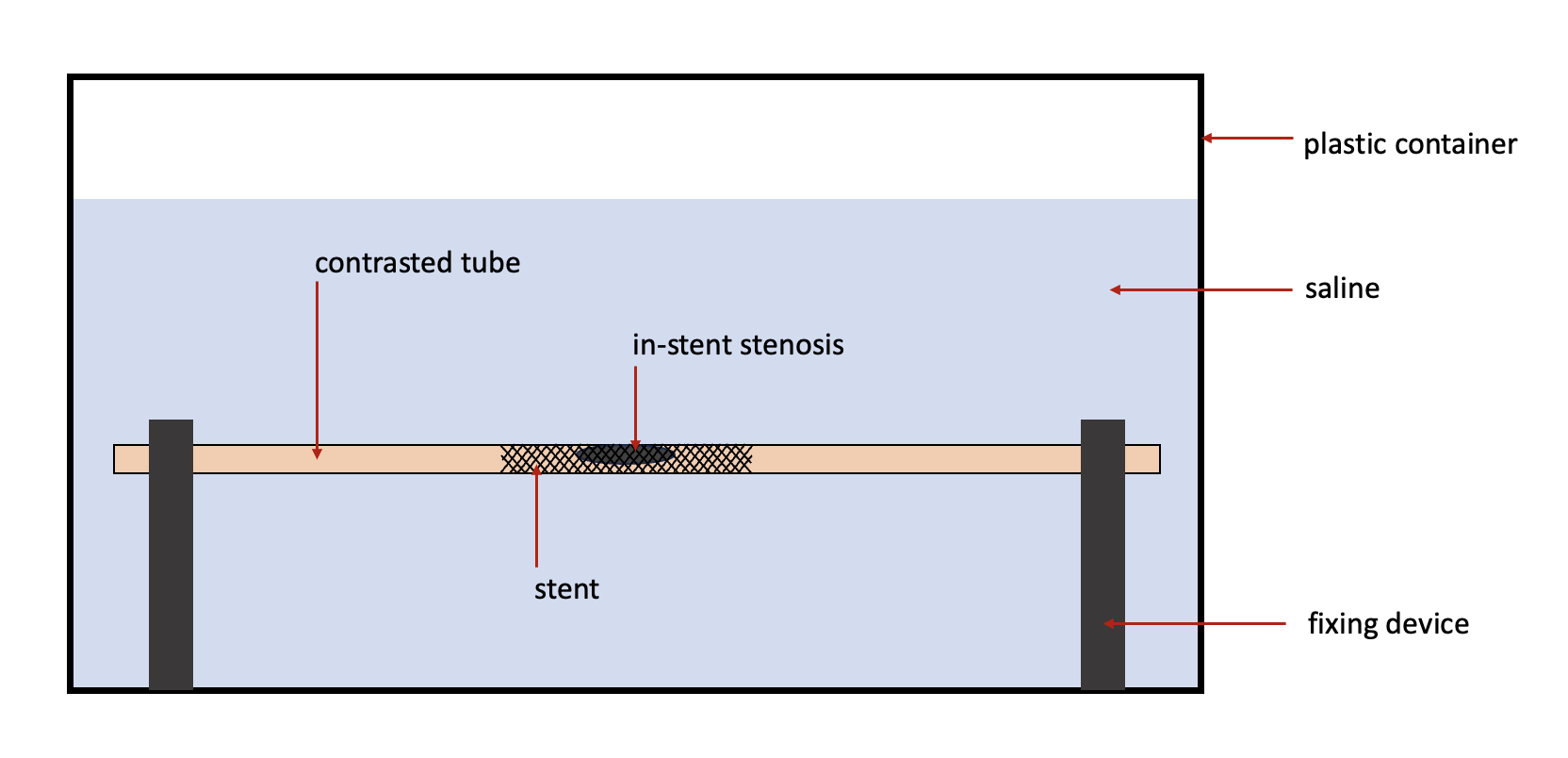


In this phantom the coronary vessel was represented by a plastic tube with a diameter of approximately 3 mm. The wall thickness of the plastic tube was about 0.3 mm with a density of 35 HU, the stents in the center of the plastic tubes. The artificial hypodense stenoses were made of a wax-based material mixed with a lipophilic contrast agent (Lipiodol Ultra-Fluid; Guerbet GmbH, Sulzbach, Germany) titrated to measure 45 HU at 120 kVp. A small portion of the wax-based material was angiographically positioned in the lumen of the stent. The material was then pressed and thus fixed to the stent strut with a 1.5-mm balloon catheter (Armada 14; Abbott GmbH, Wiesbaden, Germany) over a microwire (V-14 Control Wire; Boston Scientific GmbH, Ratingen, Germany). The tube was sealed airtight on both sides and placed in a plastic container with the help of a fixing device. The container measured (length) 36 cm × (width) 24 cm × (height) 14 cm and was filled with saline (0.9 %). This container was finally placed in the gantry of the scanner so that the plastic tube with the stent came into position slightly below the isocenter.
